# Lateral Tibial Posterior Slope Induces Anterior Tibial Translation While Lateral-Medial Slope Difference Induces Internal Rotation: A Cadaveric Biomechanical Study

**DOI:** 10.1177/03635465261480902

**Published:** 2026-09-22

**Authors:** Shuntaro Nejima, Jonathan Holt, Richard van Arkel, Andrew Metcalfe, Nicholas Smith, Andrew Amis

**Affiliations:** * Biomechanics Group, Mechanical Engineering Department, Imperial College, London, United Kingdom; † Department of Orthopaedic Surgery, Yokohama City University, Yokohama, Japan; ‡ University Hospitals Coventry and Warwickshire, Coventry, United Kingdom; Investigation performed at Imperial College, London, United Kingdom

**Keywords:** anterior cruciate ligament, posterior tibial slope, osteotomy, anterior tibial translation, internal tibial rotation

## Abstract

**Background:**

Posterior tibial slope (PTS) influences tibiofemoral kinematics and anterior cruciate ligament (ACL) function. While previous studies have evaluated symmetrical slope modifications, the isolated influences of the medial or lateral PTS remain unclear. This study investigated the influence of medial and lateral PTS changes on anterior tibial translation (ATT) and internal rotation (IR) under axial loading, using unicondylar slope-changing osteotomies.

**Hypothesis:**

Increased lateral PTS increases ATT, while a greater delta PTS (lateral minus medial PTS) increases IR.

**Study Design:**

Controlled laboratory study.

**Methods:**

Eight fresh-frozen knee specimens were mounted in a custom-built fixture in a compression/torsion loading machine. Tibiofemoral translation/rotation kinematics were measured under a 500-N axial compression load at 0° and 20° of knee flexion using optical motion tracking. Unicondylar tibial slope-changing osteotomies were performed on the medial and lateral tibial plateaus with a custom cutting guide, preserving key ligament and meniscus root attachments. Medial and lateral PTS were independently adjusted to −5°, 0°, +5°, and +10°, resulting in medial and lateral PTS ranging from −5° to +10°, using 3-dimensionally printed wedges. Repeated-measures analyses of variance, post hoc *t* tests, and Pearson correlations were used for analysis.

**Results:**

An increase of +10° in lateral PTS significantly increased ATT (*P* = .012), while increasing medial PTS did not (*P* > .99). IR was most influenced by delta PTS. At 0° of flexion, altering lateral or medial PTS alone was insufficient to significantly change rotation, but changing delta PTS +10° produced 7.6° more rotation (*P* = .015). At 20° of flexion, changing individual slopes ±10° significantly increased/decreased rotation (*P* < .036). The largest rotational differences correlated with opposing delta PTS values (*r* = 0.960; *P* = .0006).

**Conclusion:**

Lateral PTS was the principal determinant of ATT, whereas delta PTS was the principal determinant of IR under axial loading.

**Clinical Relevance:**

Understanding the independent biomechanical roles of the medial and lateral PTS provides insight into mechanisms of anterior and rotational instability in ACL injury and reconstruction. These findings suggest the potential utility of differential correction of PTS as a novel surgical approach for optimizing tibiofemoral rotational kinematics.

The posterior tibial slope (PTS) is recognized as a critical anatomic factor influencing knee biomechanics, particularly in relation to anterior cruciate ligament (ACL) injury and reconstruction. An increased PTS leads to greater anterior tibial translation (ATT), which consequently imposes higher strain on the native ACL or a reconstructed graft.^8,16,19,30,34,38,43,49^ Patients with a steeper PTS have a significantly higher risk of sustaining a primary ACL injury^10,23,42,44,54,57^ as well as graft failure after ACL reconstruction.^1,7,11,28,36,40,48,63^ Accordingly, slope-reducing osteotomy has emerged as a relevant surgical strategy to mechanically correct this predisposing anatomy in both primary and revision ACL reconstruction.^∥^

Although numerous studies have assessed medial and lateral PTS separately,^¶^ most have relied on imaging-based measurements rather than experimentally isolating the functional contribution of each plateau. As a result, the causal effects of independently altering medial and lateral PTS remain unclear. This is important because the medial and lateral tibial plateaus are anatomically distinct and play different roles in tibiofemoral kinematics.^25^ Consequently, the independent biomechanical effects of medial and lateral PTS, as well as the influence of their difference (delta PTS), on ACL strain and knee stability have not been fully elucidated.

Therefore, the aim of this study was to evaluate the independent and combined effects of medial and lateral PTS on ATT and internal tibial rotation (ITR)—both of which are known to critically increase ACL strain^32,33,41^— under axial loading, using a unicondylar tibial slope-changing osteotomy. We hypothesized that an increased lateral PTS would lead to greater ATT, while a larger delta PTS (lateral minus medial PTS) would increase ITR.

## Methods

The overall plan was to apply a 500-N load to the knee while varying the medial and lateral PTS through unicondylar slope-changing osteotomy. Measurements included the anterior-posterior translation and rotational changes of the tibia relative to the femur.

### Specimen Preparation

This study utilized 8 fresh-frozen human cadaveric knee specimens (4 left, 4 right), obtained from donors with a mean (± SD) age of 67.8 ± 2.4 years (range, 64-70 years), including 7 men and 1 woman. Using data from a similar experiment,^61^ it was determined using G*Power Version 3.4 software that translations of 2.2 mm could be identified with 80% power and 95% confidence with 8 specimens. The study received ethical approval from the Imperial College Healthcare Tissue Bank project R21053-1A. The specimens were kept at −20°C and thawed for 24 hours before testing. An orthopaedic surgeon (S.N.) confirmed the absence of osteoarthritis and ligament injury through manual palpation and visual inspection. The skin, subcutaneous tissue, patella, and posterior muscles, excluding the popliteus, were removed. The tibia and femur were cut 150 mm distal and proximal to the joint line, respectively. To maintain anatomic position, the proximal 70 mm of the fibula was fixed to the tibia using a tricortical bone screw. Soft tissues surrounding the proximal femur and distal tibia were removed, and the bone ends were cemented in 60 mm–diameter cylindrical steel pots using polymethyl methacrylate bone cement. The vertical axis of the tibial shaft was centered within the pot, aligning the compression axis of the testing apparatus to pass through the midpoint of the tibial plateau, between the tibial spines. The tibia was held in this posture by a set of pointed screws passed through the wall of the bone pot to allow accurate alignment before cementing. The femur was fixed similarly, ensuring the femoral and tibial shafts were parallel in the sagittal plane, which was defined as 0° of knee flexion. In the coronal plane, the femoral shaft (anatomic) axis was angled approximately 6° from the vertical, reflecting the divergence between the femoral mechanical and anatomic axes. As with the tibia, the femur was held and adjusted in its pot using pointed screws to ensure the desired alignment before pouring the bone cement into the femoral pot. Thus, both bone pots were mounted coaxially in the testing machine when the knee was in full (0°) extension, with the loading axis passing through the midpoint of the tibial plateau.

Radiographic images of the lateral aspect of the knee with the medial and lateral femoral condyles fully overlapping were taken using a C-arm to measure the medial and lateral PTS. The medial PTS was defined as 90° minus the angle formed by the intersection between the longitudinal axis of the tibia and the slope of the medial tibial plateau. The longitudinal axis of the tibia, which was parallel to the metal pot used to fix the tibia, was determined by a line connecting the midpoint of the anterior-posterior diameter of the tibia just inferior to the tibial tubercle and the midpoint of the anterior-posterior diameter at least 50 mm distal to the first point.^10^ The lateral PTS was measured in the same manner. The medial tibial plateau was identified as wider and concave in shape, whereas the lateral tibial plateau appeared narrower and either flat or slightly convex. Delta PTS was calculated by subtracting the medial PTS from the lateral PTS, with positive values indicating a steeper lateral slope and negative values indicating a steeper medial slope.

### Axial Compression Load in Relation to Different PTS

Axial compression loading was applied using a dual-axis servo-hydraulic testing machine (Instron 8874; Instron Co). A custom-designed fixture holding the potted femur was connected to the actuator of the moving test machine^61^ (Figure 1). This machine could also apply internal-external rotation to the femur around the long axis of the tibia. It automatically maintained zero torque during axial loading, thus measuring the coupled internal-external rotation of the tibiofemoral joint induced by the tibial slopes.

**Figure 1.**
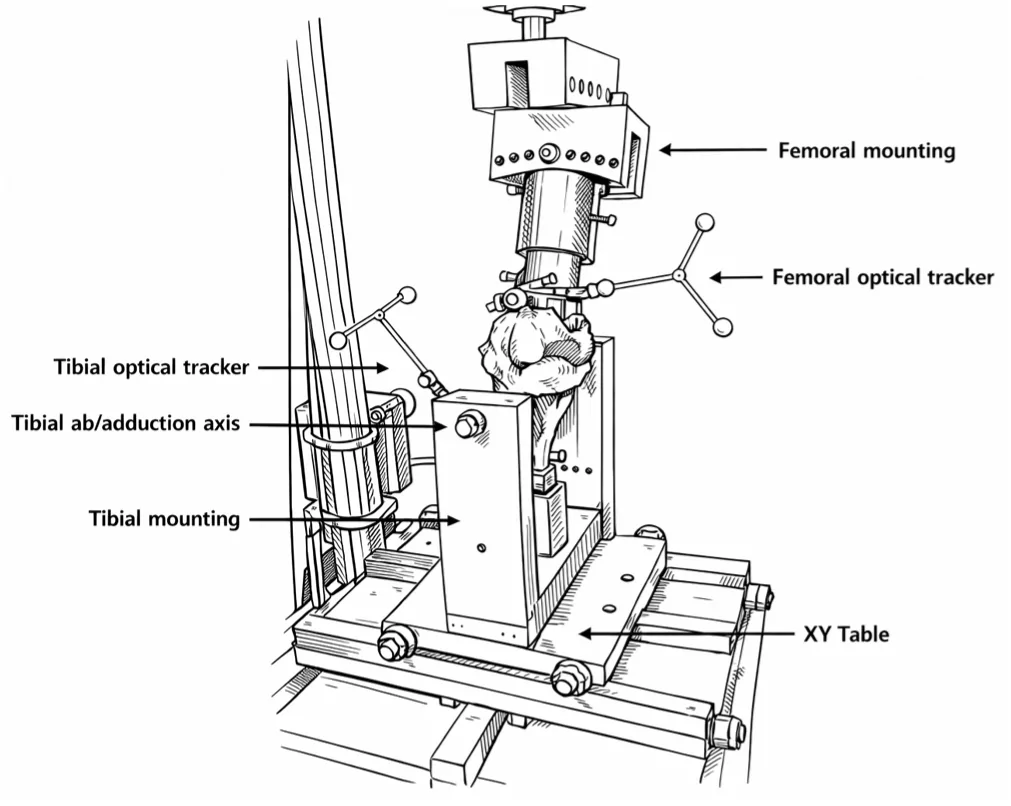
Experimental setup of a right knee specimen at 20° of knee flexion. The tibial mounting table allowed for secondary motions, such as abduction-adduction and anterior-posterior and medial-lateral translations.

This setup allowed the knee to be positioned from 0° to 20° of flexion, covering the range of motion during the stance phase of walking when the knee is most heavily loaded at heel strike and toe-off.^50^ The potted tibia was secured to a custom-designed pivoting apparatus on an XY rolling table. This allowed anterior-posterior and medial-lateral translations and rotation about the anterior-posterior abduction-adduction axis in response to the applied loads. The free rotation of the tibia around the anterior-posterior axis maintained the load distribution between the medial and lateral condyles, for example, if the femur slid down the posterior slope more on one condyle than the other. The rotary axis of the testing machine automatically adjusted the knee into an internally-externally neutral position, where the torque was zero in every test. Axial compression loads were applied to the knees, always directed vertically through the center of the tibial plateau. The baseline measurement for optical tracking began with an initial compression load of 20 N. Subsequently, the axial load was increased at 25 N/s to 500 N. This compressive load was determined through preliminary testing. Initially, a compressive load equivalent to twice the body weight—representing the force acting on the knee joint during the stance phase of gait—was intended.^50^ However, after the bi-unicondylar osteotomy, an avulsion fracture occurred at the central portion of the tibial plateau that included the tibial attachment of the ACL, at 1200 N, due to the stress concentrations at the base of the osteotomy cuts medial and lateral to the cruciate ligament attachments. This failure prompted a reduction in maximum load to 500 N. Data from the optical tracking systems were recorded at 500 N at 0° and 20° of knee flexion.

The first stage of the experiment loaded the knee with the tibia intact, before osteotomy. This confirmed that even after performing osteotomies in both the medial and lateral compartments and inserting 0° wedges—thus not altering the PTS—the knee mechanics did not change significantly.

### Kinematic Data Measurement

To measure tibiofemoral motion, a stereo camera system (Polaris Vega; Northern Digital Inc) with an accuracy of ±0.12 mm was used. Optical markers (Brainlab) were attached to the femur and tibia via bicortical rods. Small fiducial screws were placed in the femur and tibia to establish the bone coordinate system relative to the camera coordinate system. The screws in the femur were positioned at the medial and lateral femoral epicondyles and the proximal femoral end, while those in the tibia were located at the most lateral and medial points of the plateau below the osteotomy site and at the distal end of the tibia. Kinematic data were processed using MATLAB code (MathWorks) developed and validated for analyzing knee kinematics.^4^ ATT was calculated as the anterior translation of the midpoint of the digitized medial-lateral line across the tibial plateau along a “floating” axis perpendicular to the long axis of the tibia in the sagittal plane, as the load increased from 20 to 500 N, relative to the midpoint of the digitized transepicondylar axis of the femur, which did not move in anterior-posterior translation. Internal-external rotations were measured directly by the rotary actuator of the test machine as femoral rotation from the initial datum position under 20 N of compressive force to the position at which the torque induced by the PTS was zero, noting that tibial internal-external rotation was fully constrained by the X-Y table mounting.

### Unicondylar Slope-Changing Osteotomy

Unicondylar slope-changing osteotomies were performed at the medial and lateral tibial plateaus by a consultant knee surgeon (S.N.) with specialization in osteotomy. At the medial side, the superficial medial collateral ligament (sMCL) was gently elevated from the tibia at the level of the transverse osteotomy without damage. A guide wire was inserted into the medial tibial condyle between the sMCL and deep medial collateral ligament attachments at the midpoint of the tibial anterior-posterior diameter, directed perpendicular to the tibial shaft to the center of the tibia, 20 mm below the joint line. The tibia was then drilled over the guide wire using a 12 mm–diameter cannulated drill. A purpose-built cutting guide was slid into the drill hole and then fixed anteriorly with 2 wires (Figure 2A).

**Figure 2.**
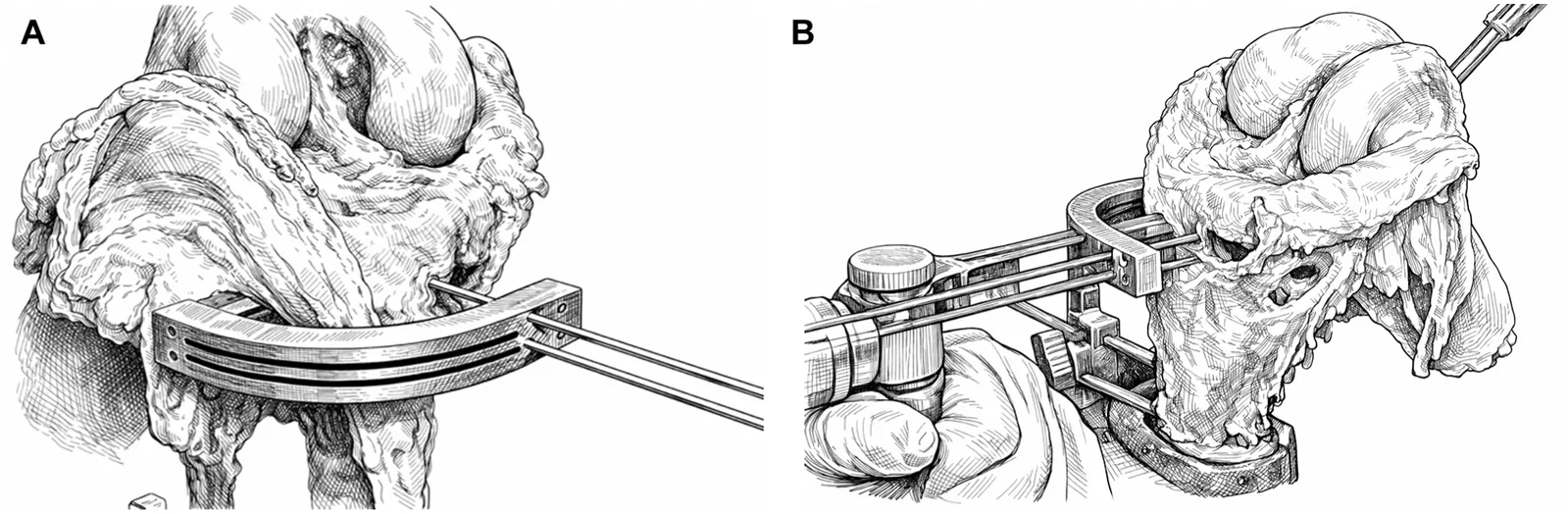
(A) The cutting guide was secured to the lateral side of the right tibia using 2 wires. (B) The bone saw was inserted through the anterior slot of the guide to perform the transverse cut for the lateral compartment in the right knee. In this specimen, the osteotomy on the medial compartment had already been performed.

The spacing between the 2 slots in the cutting guide was 5 mm, and transverse osteotomies were performed with a bone saw through these slots, with the central portion of the proximal tibia left in place (Figure 2B).

After the cutting guide was removed, longitudinal osteotomy was performed in the medial compartment with a fine chisel without damaging the anterior and posterior roots of the medial meniscus and cruciate ligaments. A 5 mm–thick bone fragment was removed. The same procedure was performed in the lateral compartment, where the transverse osteotomy was proximal to the tibiofibular joint, preserving the attachment of the iliotibial band.

Three-dimensionally printed nylon wedges with 5 different angles (−10°, −5°, 0°, +5°, and +10°) were created, including a 12 mm–diameter cylinder (Figure 3). By inserting these wedges into the medial and lateral osteotomy sites, medial (M) and lateral (L) PTS were independently adjusted to −5°, 0°, +5°, and +10°, resulting in medial and lateral PTS ranging from −5° to +10°, with minimal change in height at the center of each tibial plateau (Figure 4). This minimized the effect of the change in PTS on the coronal alignment of the knee and the ligament tensions. In addition, the interlocking of the cylinder with the bone fragments above and below the transverse osteotomy ensured that the proximal fragment remained located correctly on the shaft of the tibia, and no movement of the plateau fragment was observed.

**Figure 3.**
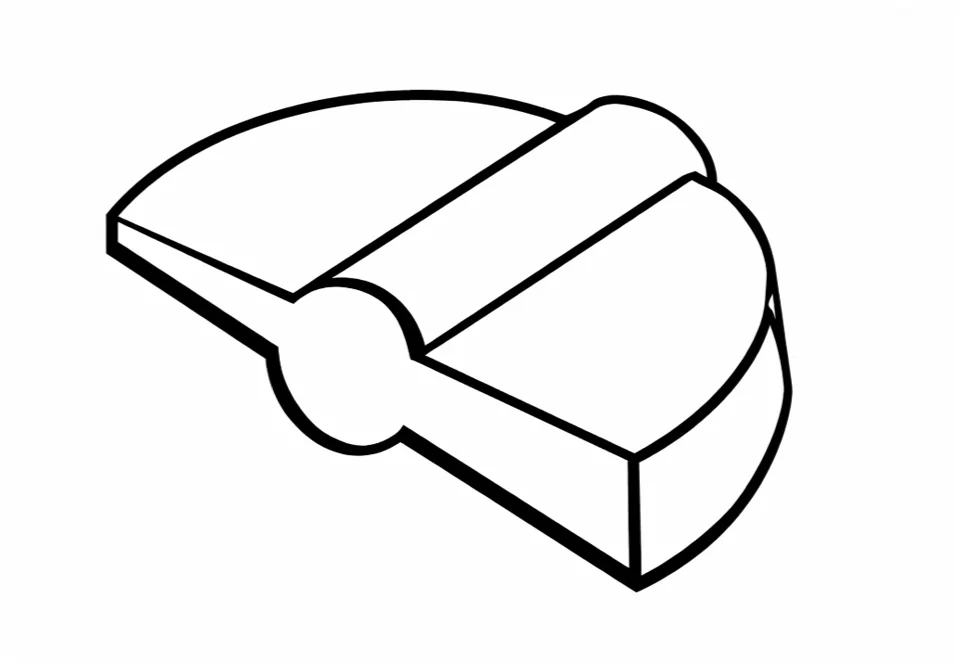
Schematic of a 3-dimensionally printed slope-changing insert. It has a 12 mm–diameter cylinder aligned on a medial-lateral axis to preserve the positions of the bone fragments postosteotomy. The curved shape of the areas anterior and posterior to the cylinder was customized to match the outline of the tibia.

**Figure 4.**
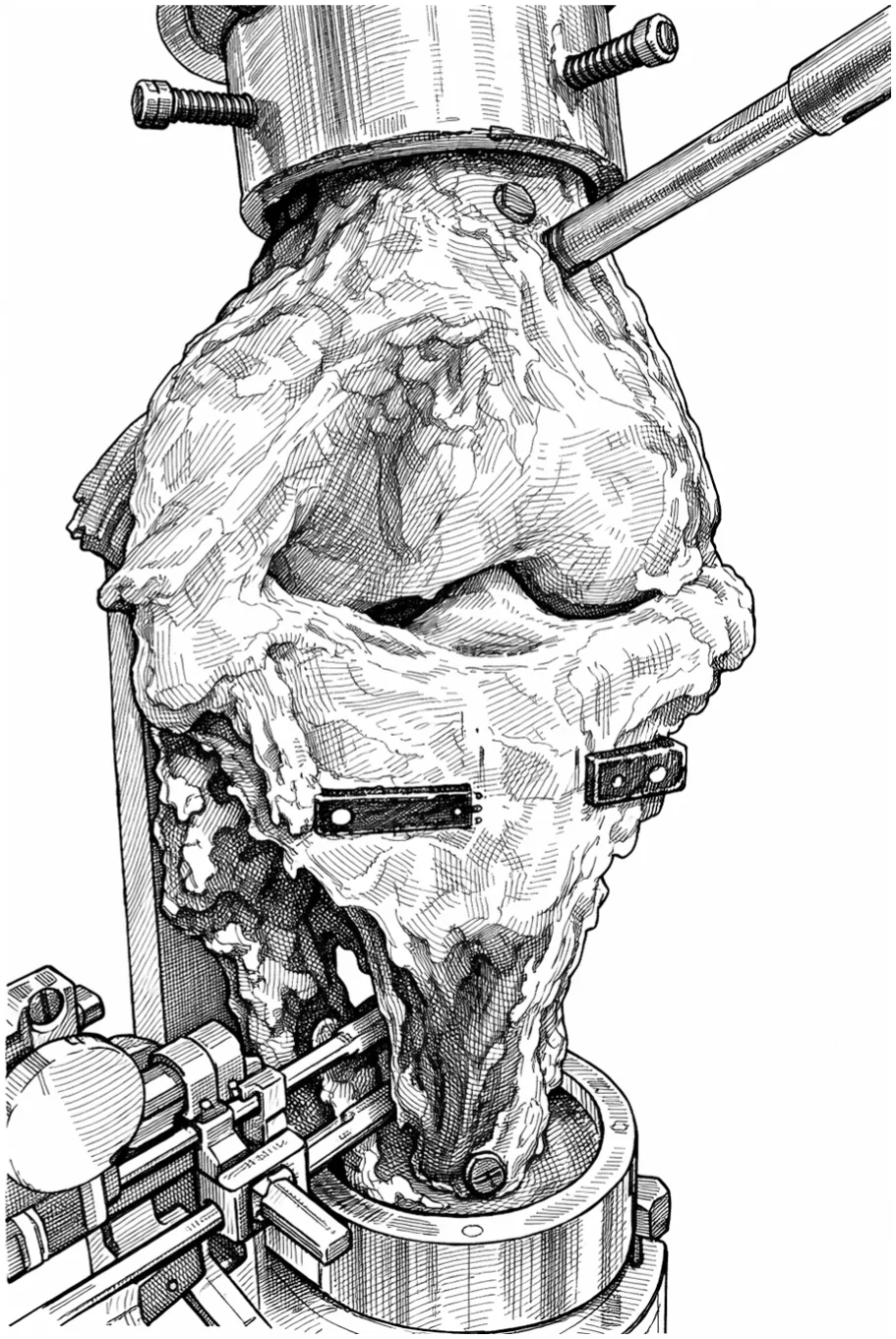
By inserting 3-dimensionally printed wedges medially and laterally, the posterior tibial slope could be independently adjusted on the medial and lateral plateaus. The central portion of the tibia, the tibial anterior cruciate ligament attachment site, was preserved. In this right knee, a 0° wedge is placed laterally and a +10° wedge medially.

After slope-changing osteotomy of each compartment, tibiofemoral movements under load were measured at each combination of the medial and lateral PTS at 0° and 20° of flexion. Measurements were taken with the following combinations of lateral and medial PTS changes: (0°, 0°), (+10°, 0°), (0°, +10°), (+5°, −5°), (−5°, +5°), (+5°, +5°), and (−5°, −5°) in addition to the intact state. There should be no difference of tibial motion between the intact knees and those with 0° wedges inserted on both sides. Tibiofemoral kinematics were represented using the tibial position under a 20-N compression load at 0° of knee flexion in the intact condition as the reference, with ATT and IR defined as positive values.

### Statistical Analysis

The data were analyzed using SPSS Version 28.0 for Windows (IBM Corp). *P* values <.05 were considered significant. The Shapiro-Wilk test was used to check that the data distributions were appropriate for parametric analysis. The Pearson correlation coefficient was used to examine the correlation between the lateral PTS, medial PTS, and delta PTS with tibiofemoral movements in intact knees. Repeated-measures 2-way analyses of variance were used to determine whether there were significant differences between groups in tibiofemoral movements with independent variable combinations of lateral and medial PTS changes: intact state, (0°, 0°), (+10°, 0°), (0°, +10°), (+5°, −5°), (−5°, +5°), (+5°, +5°), and (−5°, −5°) and knee flexion angle (0° or 20°). If there were significant differences, post hoc paired *t* tests with Bonferroni correction were utilized.

## Results

The native mean medial PTS, lateral PTS, and delta PTS were 9.1°± 2.3° (range, 5.6° to 12.2°), 12.5°± 3.5° (range, 6.5° to 17.0°), and 3.5°± 4.0° (−1.4° to 11.4°), respectively. When the native knee flexed 20°, the mean calculated movements from the zero datum at 0° of flexion were an ATT of 8.6 ± 3.0 mm (range, 4.2 to 11.9 mm) (femoral rollback) and an IR of 11.4°± 2.3° (range, 6.1° to 13.3°).

### ATT Under a 500-N Compression Load

There was no significant interaction between wedge combination and knee flexion angle (*P* = .074), indicating that the effect of wedge combination on ATT was consistent between 0° and 20° of flexion (Figure 5). Across all wedge combinations, ATT at 20° of flexion was significantly greater than that at 0° (overall *P* < .001). When pooled across conditions, the mean ATT was 0.7 ± 1.1 mm (range, −3.9 to 2.6 mm) at 0° and 8.4 ± 3.3 mm (range, −2.6 to 12.7 mm) at 20°.

**Figure 5.**
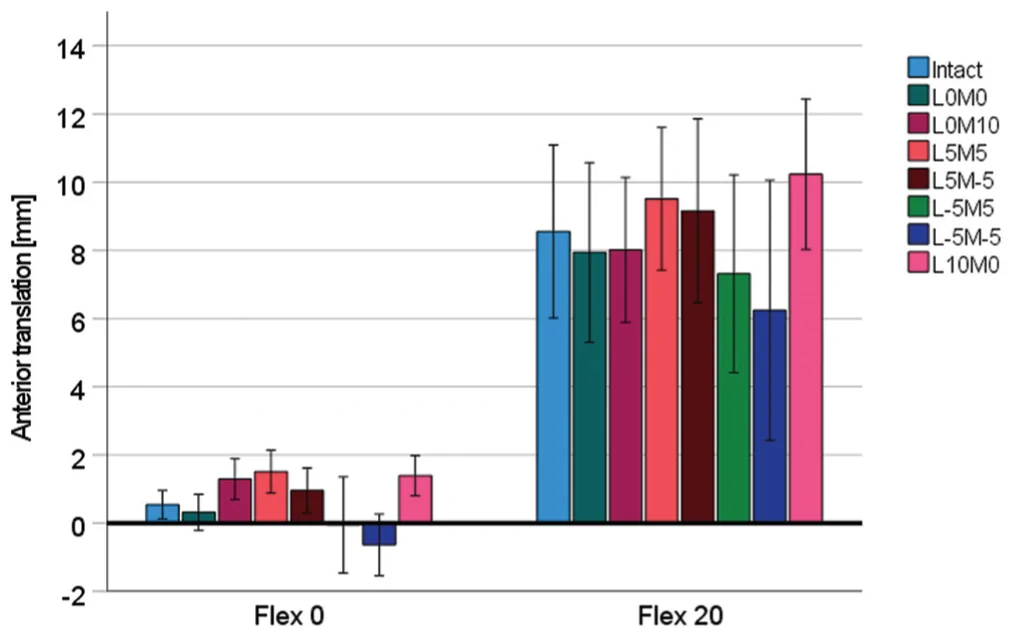
Anterior tibial translation at 0° and 20° of knee flexion under a 500-N compression load (mean ± SD; n = 8). The values −5, 0, 5, and 10 are the change in the posterior slope from the native value (degrees). L, lateral slope; M, medial slope.

There was no difference in ATT between the intact condition and L0M0 (*P* > .99), indicating that the osteotomy procedure itself did not affect tibial translation.

Increasing the medial PTS alone did not change the ATT (L0M0 vs L0M10; *P* > .99). Significant correlations were not found between medial PTS and ATT at 0° of flexion (*r* = 0.423; *P* = .344) and 20° of flexion (*r* = 0.091; *P* = .847).

In contrast, increasing the lateral PTS led to greater ATT, as shown by the higher values at L10M0 compared with L0M0 (difference, 1.7 mm; *P* = .012). Similar findings were observed in the combined wedge conditions, with ATT significantly greater at L5M-5 than at L-5M-5 (difference, 2.3 mm; *P* = .036). Significant correlations were found between lateral PTS and ATT at 0° of flexion (*r* = 0.8376; *P* = .0187) and 20° of flexion (*r* = 0.966; *P* = .0004) (Figure 6). The magnitudes of the translations reflect the knee being tighter at 0° of flexion than at 20°.

**Figure 6.**
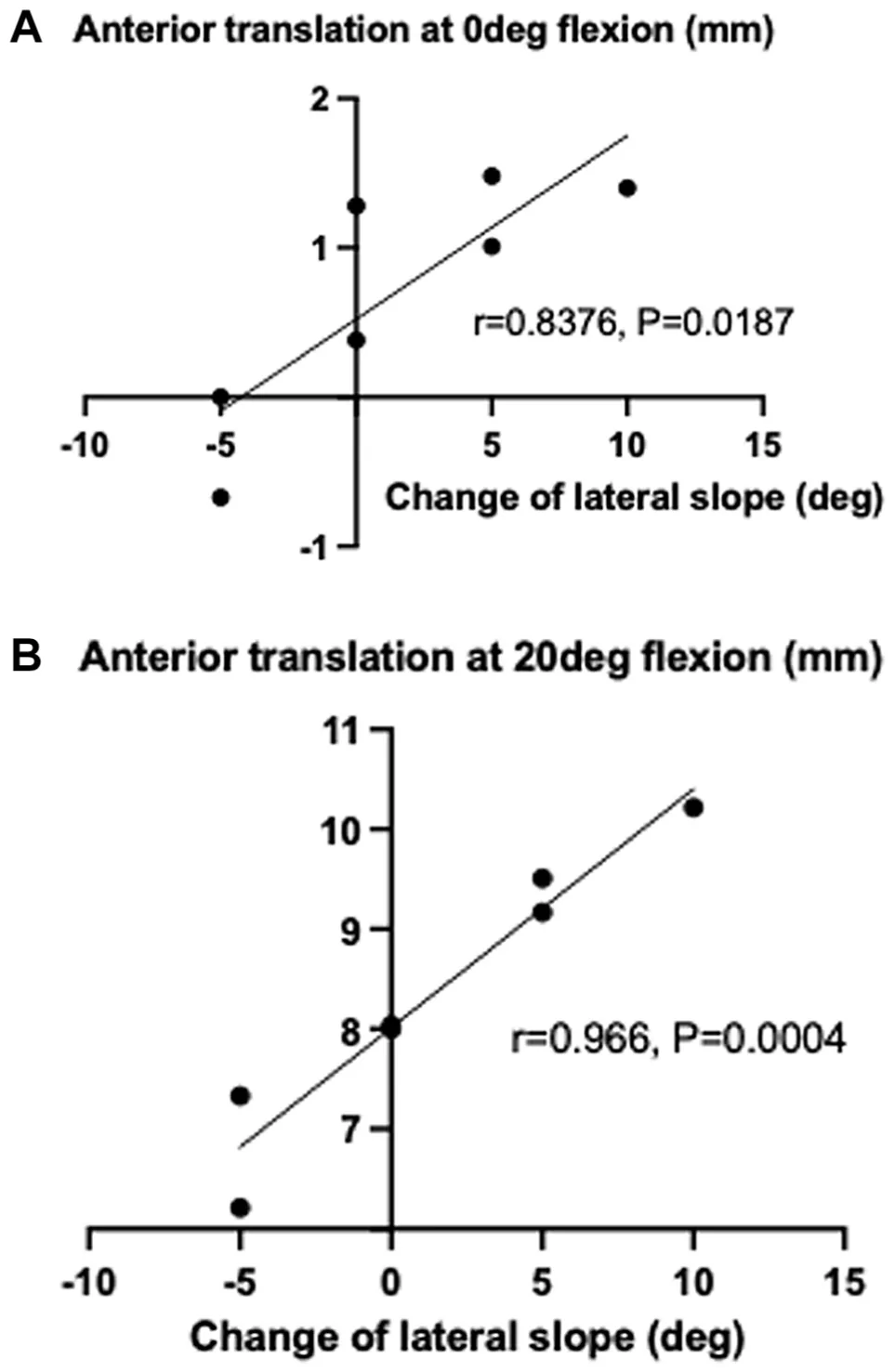
Significant correlations were found between lateral PTS and ATT at (A) 0° of flexion (*r* = 0.8376; *P* = .0187) and 20° of flexion (*r* = 0.966; *P* = .0004). Each data point is the mean of 8 knees. There are 2 data points at 8 mm of ATT at 20° of flexion.

### Internal Rotation Under a 500-N Compression Load

There was a significant interaction between wedge combination and knee flexion angle (*P* = .005) (Figure 7). Across all wedge combinations, IR at 20° of flexion was significantly greater than that at 0°, indicating that increasing knee flexion from 0° to 20° induced internal rotation.

**Figure 7.**
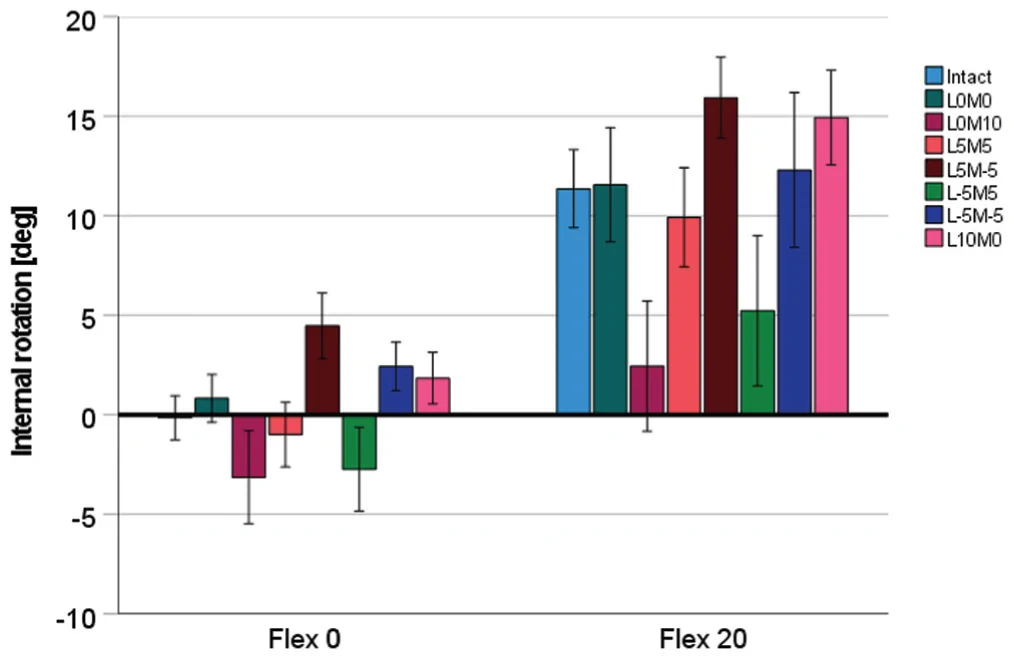
Internal rotation at 0° and 20° of knee flexion under a 500-N axial compression load (mean ± SD; n = 8). The values −5, 0, 5, and 10 are the change in the posterior slope from the native value (degrees). L, lateral slope; M, medial slope.

At both 0° and 20° of flexion, there was no difference in IR between the native condition and L0M0 (*P* = .45 at 0°; *P* > .99 at 20°), indicating that the osteotomy procedure itself did not affect IR.

At 0° of flexion, altering either the medial PTS alone (L0M10 vs L0M0; *P* > .99) or the lateral PTS alone (L10M0 vs L0M0; *P* = .45) did not significantly affect IR. In contrast, wedge combinations that produced opposing delta PTS values generated substantial rotational differences. L5M-5 produced 7.6° more IR than L0M10 (*P* = .015) and 7.2° more IR than L-5M5 (*P* = .018). A significant correlation was found between delta PTS and internal rotation (*r* = 0.8826; *P* = .0085) (Figure 8A). These findings indicate that delta PTS, rather than isolated changes in medial or lateral slope, was the primary determinant of IR in full extension.

**Figure 8.**
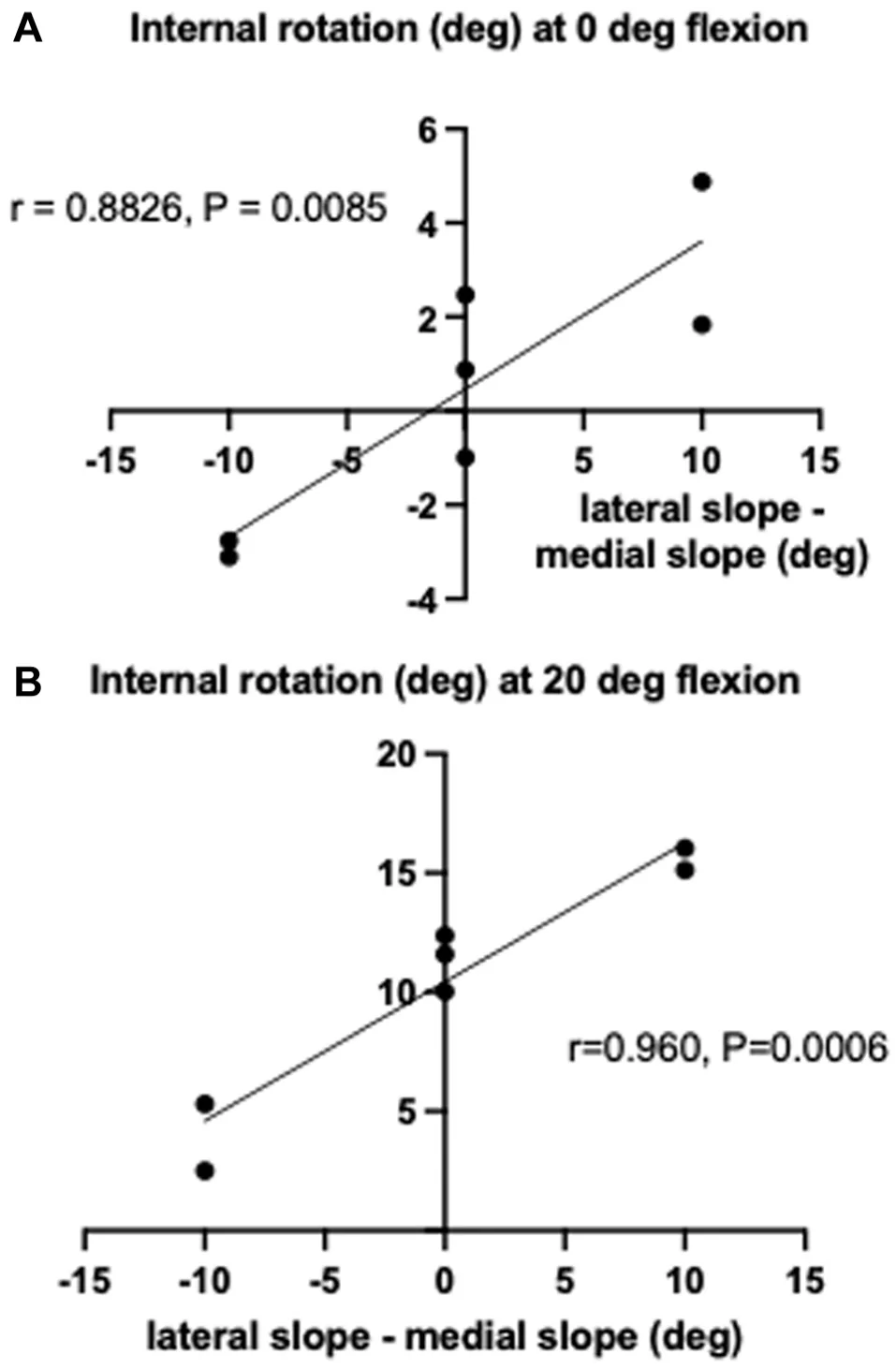
Significant correlation was found between delta posterior tibial slope and internal rotation at (A) 0° of flexion (*r* = 0.8826; *P* = .0085) and (B) 20° of flexion (*r* = 0.960; *P* = .0006). Each data point is the mean of 8 knees.

At 20° of flexion, the influence of delta PTS was amplified. Increasing lateral PTS alone increased IR (L10M0 vs L0M0, +3.4°; *P* = .036), whereas increasing medial PTS alone reduced it (L0M10 vs L0M0, −9.1°; *P* = .032). The largest rotational differences were observed between wedge combinations with opposing delta PTS values: L5M-5 generated 13.5° more IR than L0M10 (*P* = .003) and 10.7° more than L-5M5 (*P* = .003), and L10M0 produced 12.5° more IR than L0M10 (*P* = .006). A significant correlation was found between delta PTS and internal rotation at 20° of flexion (*r* = 0.960; *P* = .0006) (Figure 8B).

Overall, these findings indicate that tibial IR is influenced primarily by delta PTS, rather than by either plateau in isolation, and that this influence is more pronounced at 20° than at 0° of flexion.

## Discussion

This study investigated the independent and combined biomechanical effects of medial and lateral PTS on ATT and IR when the knee was loaded using a novel unicondylar slope-changing osteotomy model. The most important finding was that ATT and IR were governed by different aspects of tibial slope geometry. Specifically, lateral PTS was the principal determinant of ATT, whereas delta PTS was the principal determinant of IR.

Numerous in vivo studies that evaluated medial and lateral PTS separately have suggested that an increased lateral PTS, but not medial PTS, is associated with a higher risk of ACL injury and graft failure after reconstruction.^13,17,20,22,27,46,56,59^ In addition, Edwards et al^17^ reported that delta PTS is an independent risk factor for pediatric ACL injury. More recently, Ayuob et al^5^ also demonstrated that delta PTS is an independent risk factor for ACL reconstruction failure in a large clinical cohort. Furthermore, both lateral PTS and delta PTS have been linked to the severity of the pivot shift in ACL-injured knees, underscoring their relevance to rotational instability.^26,45,53^ These relationships are biomechanically plausible and supported by prior literature. Shultz and Schmitz^51^ reported that a lower medial-to-lateral slope ratio was associated with greater knee internal rotation excursions during landing tasks. Greater lateral-to-medial slope asymmetry has also been associated with increased dynamic IR during land-and-cut tasks, reinforcing its contribution to high-risk knee kinematics relevant to ACL injury mechanisms.^37^ In the same cohort, a steeper lateral PTS was independently related to greater anterior tibial reaction forces.^37^ Cadaveric study findings further demonstrate that, under compressive loading, lateral slope and slope differential exert a predominant influence on IR in ACL-intact knees.^60^ These findings are consistent with those of the present study; however, their observational or modeling-based designs do not allow causal relationships to be established. By experimentally altering medial and lateral PTS independently, the present study provides direct biomechanical evidence that lateral PTS is the primary determinant of ATT, whereas delta PTS governs IR under axial loading.

Lateral PTS was the dominant determinant of ATT, with medial PTS exerting minimal influence in this study. This reflects the anatomic convexity of the lateral plateau, which allows greater anterior shear under axial loading.^25^ Crucially, the impact of PTS on ATT did not depend on knee flexion angle. Although ATT was slightly greater at 20° than at 0°, the absence of a significant interaction indicates that lateral PTS governs anterior tibial shear in a flexion-independent manner near knee extension, although lateral condylar rollback might influence ATT more strongly in deeper flexion.

Tibial IR was highly sensitive to the asymmetry between medial and lateral PTS. A larger delta PTS consistently increased IR, consistent with the stable, concave medial plateau acting as a pivot and the more mobile lateral plateau permitting rotation.^25^ Importantly, a significant interaction indicated that the rotational effect of slope asymmetry was amplified at 20° of flexion. In full extension, rotational freedom is limited and the influence of delta PTS is modest, whereas early flexion increases rotational mobility through posterior rolling of the lateral femoral condyle, allowing slope asymmetry to exert a much larger effect. This mechanistic relationship provides a plausible explanation for why rotational laxity and the pivot shift are most pronounced in slightly flexed knee positions.^18^

Slope-reducing osteotomy has been shown to decrease ATT and thereby reduce ACL graft forces,^3,24,62^ making it an effective procedure in revision ACL reconstruction and, in some cases, even in primary reconstruction when a markedly increased PTS is present.^#^ In addition to its effect on anterior translation, the rotational consequences of tibial slope have also been explored in biomechanical modeling. Marouane et al^35^ demonstrated that increasing the overall tibial slope led to greater internal rotation during the stance phase of gait simulations. Amirtharaj et al^3^ reported that slope-reducing osteotomy did not substantially alter IR under isolated axial compression but decreased IR when axial compression was combined with valgus torque, suggesting that rotational effects may become more apparent under complex loading conditions. Conversely, other studies have reported that altering overall tibial slope has minimal effect on IR,^19,24^ leaving the rotational consequences of conventional bicondylar slope correction incompletely understood. In the present study, comparisons between wedge conditions that decreased the overall PTS (eg, L0M0 vs L5M5, L-5M-5 vs L0M0, and L-5M-5 vs L5M5) showed that it did not reduce IR, indicating that global slope reduction alone is insufficient to meaningfully alter rotational behavior under axial loading. Instead, our findings demonstrate that delta PTS is a much stronger determinant of tibial IR. These observations suggest that an asymmetric slope correction, reducing the lateral slope by more than the medial slope, or a lateral unicondylar slope-reducing osteotomy, may offer a more direct and effective means of correcting rotational instability by simultaneously reducing ATT through lateral slope correction and improving ITR through normalization of delta PTS. Further studies are required to determine whether unicondylar slope-changing osteotomy can be translated into clinical practice. That work might include developing novel surgical instrumentation and testing under more physiological conditions such as cyclic loading at higher forces than in the present study.

Understanding the independent biomechanical roles of the medial and lateral PTS provides insight into mechanisms of anterior and rotational instability in ACL injury and reconstruction. These findings suggest the potential utility of unicondylar tibial slope-changing osteotomy as a novel surgical approach for optimizing tibiofemoral kinematics, particularly in cases of ACL reconstruction with rotational instability.

### Limitations

The findings of this study should be interpreted in the context of several limitations inherent to an in vitro design using cadaveric specimens. First, the knees were tested under passive axial loading conditions, which do not account for dynamic muscle forces that may influence the relative contributions of the medial and lateral tibial plateaus. In particular, quadriceps action during gait will load the patellar tendon, which has an anteriorly acting tension component that will cause ATT near knee extension. This experiment was planned to show the effect of the PTS alone; therefore, it was designed to eliminate variables other than those being studied. The addition of muscle tensions acting on the cadaveric specimens might have changed the experiment toward a more physiological simulation, but the extensor mechanism is itself variable between knees, so adding muscle tensions would tend to obscure the role of the PTS. Second, the specimens were older than the population typically affected by ACL injury. Although each knee was carefully screened to exclude degenerative or structural pathology, age-related changes in tissue properties may have affected the biomechanical response and led to the load being limited to 500 N after double-compartment osteotomies. Third, the tibiae were aligned in a neutral coronal alignment. Clinical populations often present with varus or valgus deformities, and such malalignment may alter the relative impact of medial and lateral PTS on ATT or rotational laxity. Therefore, caution is warranted when extrapolating these findings to malaligned knees. This work did not simulate deeper knee flexion angles, at which unicompartmental alterations of tibial plateau height might alter the varus-valgus alignment or roll-back kinematics of the flexed knee. Finally, the loading protocol involved a 500-N axial compression force. Although this magnitude was determined through preliminary testing, and limited by avulsion of the ACL from between the medial and lateral compartment osteotomies, it was lower than the joint forces encountered during athletic activities. A larger joint force may have caused larger tibiofemoral movements. Conversely, by working in vitro, it was possible to make comparative measurements with many PTS values, including bi-unicondylar osteotomies, in each knee.

Clinical studies of PTS and its reduction, which is known to reduce the tension induced in the ACL, have usually relied on lateral-medial radiography. That method is inaccurate for measuring tibial rotations due to the variable tibial geometry^6^ but has the strength of showing the tibial translations of the medial and lateral compartments.^29^ Data from the present study could be used to estimate the motions of each compartment, based on typical anatomic intercondylar width measurements.

## Conclusion

Lateral PTS was the principal determinant of ATT, whereas delta PTS was the principal determinant of ITR under axial loading.

## Notes

∥ References 2, 12, 14, 15, 31, 47, 52, 55, 58.

¶ References 9, 13, 21, 22, 29, 39, 44, 46, 53.

# References 2, 12, 14, 15, 31, 47, 52, 55, 58.
